# Enhancing the sensitivity of gamma-aminobutyric acid and glutamate biosensors by electrochemically roughening platinum microelectrodes

**DOI:** 10.3389/fnins.2025.1679591

**Published:** 2025-09-19

**Authors:** Musefiu Yemi Adediji, Sanjeev Billa, Shabnam Siddiqui, Prabhu U. Arumugam

**Affiliations:** ^1^Institute for Micromanufacturing (IfM), Louisiana Tech University, Ruston, LA, United States; ^2^Center for Biomedical Engineering and Rehabilitation Science (CBERS), Louisiana Tech University, Ruston, LA, United States

**Keywords:** GABA, glutamate, electrochemical biosensors, platinum microelectrodes, electrochemical roughening, neurotransmitters, porous electrodes, electrochemical impedance spectroscopy

## Abstract

Gamma-aminobutyric acid (GABA) and glutamate (GLU) are two key neurotransmitters (NTs) in processing, plasticity, memory, and network functions. High-sensitivity GABA and GLU biosensors are crucial for investigating the dysregulation of the GABA-GLU balance and improving animal models and human therapies for multiple neurological disorders. We have developed a novel biosensor that utilizes electrochemically roughened (ECR) platinum (Pt) microelectrodes to achieve the highest sensitivity in detecting hydrogen peroxide (H_2_O_2_), which serves as the detection signal for the biosensors. We evaluated three microelectrode surface activation techniques—alcohol cleaning, electrochemical cleaning, and ECR—and the main effects of the ECR pulses at varying frequencies (150–6,000 Hz) on biosensor sensitivity. ECR-treated microelectrodes reveal a non-linear relationship between the pulse frequency and the H_2_O_2_ adsorption, providing the highest sensitivity. Each frequency altered the microelectrode’s roughness differently, resulting in unique surface morphologies and pore geometries, as well as the formation of surface impurities within the pores. The primary factors influencing Pt’s electrocatalytic activity are the pore geometry and the facile Pt kinetics, and not the electroactive area or the impurities in the pores. Particularly, pore geometries at low (250 Hz) and high frequencies (2,500 Hz) contribute to the highest H_2_O_2_ adsorption and sensitivity (6,810 ± 124 nA μM^−1^ cm^−2^), the highest value reported in the literature. The EIS model reveals that ECR-treated microelectrodes exhibit heterogeneous pores and partially smooth, flat regions between the pores, with the catalytic activity primarily occurring in the pore walls rather than the flat regions. The EIS data indicate superior electrical conductivity in the pore walls, which enhances the GABA and GLU peak sensitivities to 45 ± 4.4 nA μM^−1^ cm^−2^ and 1,510 ± 47.0 nA μM^−1^ cm^−2^, respectively. The corresponding limits of detection are 1.60 ± 0.13 nM and 12.70 ± 1.73 nM (*n* = 3). These findings underscore the significance of ECR in enhancing the performance of Pt MEA-based enzymatic biosensors, thereby paving the way for advanced, ultrasensitive biosensors for neurochemical monitoring in challenging *in vivo* applications.

## 1 Introduction

Neurotransmitters (NTs) like gamma-aminobutyric acid (GABA) and glutamate (GLU) are essential for maintaining the excitatory-inhibitory (E/I) balance in the central nervous system (Robinson and Jackson, 2016; Boddum et al., 2016). GABA is the primary inhibitory NT, regulating synaptic activity and preventing hyperexcitability (Boddum et al., 2016; Bhat et al., 2010), while GLU is the primary excitatory NT involved in synaptic plasticity, learning, and memory (Robinson and Jackson, 2016; Burmeister et al., 2013). Ultrasensitive detection of these two key NTs is crucial for understanding both normal neurological functions and dysfunctions, especially in conditions such as epilepsy, various neurodegenerative diseases, and addiction (Sandberg and Garris, 2010; Auteri et al., 2015; Willuhn et al., 2010; Porges et al., 2021).

Several analytical techniques have been used for NT detection, including high-performance liquid chromatography with electrochemical detection (Caudill et al., 1982; Monge-Acuña and Fornaguera-Trías, 2009; Rowley et al., 1995), and microdialysis-based biosensors (Kehr and Ungerstedt, 1988; Billa et al., 2022; Doughty et al., 2020). While these methods provide high sensitivity, they are typically offline techniques that often lack the spatiotemporal resolution necessary for real-time monitoring of rapid fluctuations in NT levels (Hugo Cifuentes Castro et al., 2014; Robinson et al., 2008). Microdialysis, which is currently considered the gold standard for NT detection, utilizes relatively large probes (a few hundred microns in diameter). This can cause damage to neuronal tissues and lead to inflammation (Hugo Cifuentes Castro et al., 2014; Robinson et al., 2008). In contrast, electrochemical biosensors, especially those based on microelectrode arrays (MEAs), have emerged as a promising alternative. They offer rapid, real-time detection of NTs with high spatial (a few tens of microns) and temporal (sub-second) resolution (Cahill et al., 1996; Tan et al., 2022; Ivanovskaya et al., 2018; Hossain et al., 2018).

Enzymatic biosensors are routinely used to detect non-electroactive NTs such as GABA and GLU. Platinum (Pt) MEAs are widely used in the development of the enzymatic biosensors due to their excellent electrocatalytic activity, high electrical conductivity, corrosion resistance, sensitivity, selectivity, stability, and biocompatibility (Ben-Amor et al., 2014; Burmeister et al., 2020). They are an ideal electrode material for *in vitro* and *in vivo* NT sensing. Among the various techniques for detecting NTs, amperometry is particularly effective because it can achieve very high time resolution with a sampling rate in the kHz range. Amperometry can capture signals on the sub-second timescale, which helps minimize interference from adsorption effects. Furthermore, the method gathers and analyzes extensive signal datasets, showing very low limits of detection (LODs) (Wu et al., 2022). In enzymatic biosensors, the concentrations of GABA and GLU are measured by detecting the oxidation currents of hydrogen peroxide (H_2_O_2_), when a constant potential of +0.7 V (Ag/AgCl) is applied to the Pt microelectrode. Here, H_2_O_2_ serves as the detection signal (Wang et al., 2024), and its current is proportional to the amounts of GABA or GLU that have been enzymatically metabolized by the immobilized enzymes cross-linked to the Pt working microelectrode (WE) (Moldovan et al., 2021). As reported by our group and others (Doughty et al., 2020; Hossain et al., 2018; Moldovan et al., 2021), GABA and GLU biosensors are coated with immobilized glutamate oxidase (GOx) for the detection of GLU, and with both GOx and GABA aminotransferase (GABASE) for the detection of GABA, using a bovine serum albumin (BSA) and glutaraldehyde matrix solution. In the GLU biosensor, GOx converts GLU into H_2_O_2_, oxidizing and releasing two electrons, generating a current signal. Similarly, in the GABA biosensor, *α*-ketoglutarate facilitates the breakdown of GABA through the action of both GOx and GABASE, converting it into GLU. This GLU is subsequently converted into H_2_O_2_, which oxidizes and produces the current signal. The current generated at each biosensor site is directly proportional to the concentrations of GABA and GLU.

Developing state-of-the-art biosensors for long-term, real-time *in vivo* detection requires overcoming several challenges. One significant challenge is the deterioration of the current detection signal and the accompanying loss of sensitivity caused by electrode surface fouling and enzyme degradation over time (Dash et al., 2009). A comprehensive approach that combines materials science, electrochemical engineering, and neuroscience is essential for developing those reliable electrochemical biosensors for animal research (short-term goal) and pre-clinical and clinical applications (long-term goals). In this work, our goal is to achieve a higher initial sensitivity before *in vivo* implantation. This is crucial because biosensors lose sensitivity during the first few days following surgery and implantation in animals (Rutherford et al., 2007). Consequently, these biosensors may not operate at full functionality for several weeks.

To overcome this poor sensitivity challenge, researchers have modified Pt microelectrodes using nanomaterials, such as carbon nanotubes and gold nanoparticles, or used surface activation techniques (Ammam and Fransaer, 2010). For example, the neurotransmitter dopamine detection limits were improved using Ce-doped ZrO₂ nanoparticle–modified electrodes (Harshavardhan et al., 2022; Matt et al., 2019; Shivakumar et al., 2021). These methods activate the Pt microelectrodes, significantly improving their electrical conductivity and electroactive surface area, enhancing sensitivity. Several surface activation methods have been explored, including solvent-based, electrochemical, and polishing (ECC, ECP), as well as electrochemical roughening (ECR) (Tan et al., 2022; Ivanovskaya et al., 2018; Moldovan et al., 2021). Each of these techniques influences biosensor performance metrics differently. ECC is a widely used method that cleans the Pt electrode surface electrochemically by removing impurities through oxidation and reduction during potential cycling. ECC increases the electrochemically active surface area and can cause mild restructuring of the Pt surface, depending on the specific parameters used (Moldovan et al., 2021). In contrast, ECR-treated Pt is particularly promising, resulting in a highly robust and homogeneous porous surface. This treatment significantly improves electrical conductivity, electron transfer kinetics, and electrocatalytic activity by promoting the dissolution and redeposition or recrystallization of platinum oxide layers, as demonstrated in this work and by others (Tan et al., 2022; Ivanovskaya et al., 2018).

**Figure 1:**
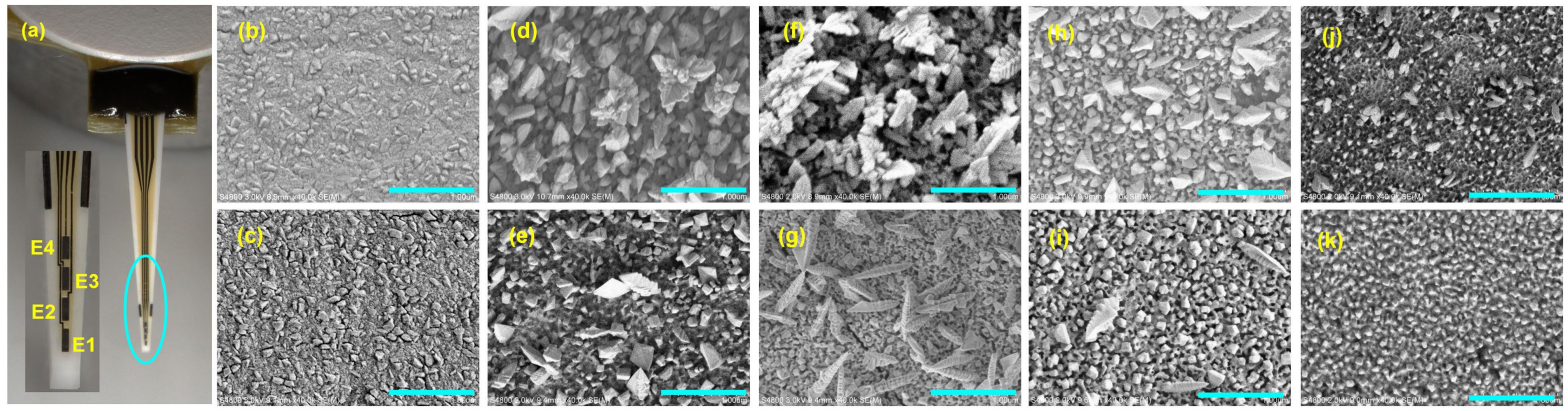
**(a)** Optical microscopy image of an R1 probe with four Pt MEAs (E1-E4) patterned linearly. Each microelectrode is 50 μm × 150 μm and is spaced 50 μm apart. SEM images of **(b)** alcohol treated, **(c)** ECC-treated, **(d–k)** +1.4 V/−0.25 V ECR pulse-treated at 150, 250, 350, 750, 1,500, 2,500, 4,000, and 6,000 kHz, respectively. The scale bar is 1 μm. The ECR-treated surfaces consist of units of heterogeneous pores that function together as a single uniform microstructure with no distinct regions of electrochemical behavior. Each frequency creates a distinct pore shape, which non-proportionately affects conductivity differences. At *lower frequencies* **(d–f)**, fractalization of grains occurs most significantly, with enhanced heterogeneity in both the flat surface and the pore walls, thereby increasing the conductivity of the pores. At *higher frequencies* **(g–i)**, fractalization reduces, impedance increases, with smoother grains causing a higher impedance than heterogeneous grains.

For the first time, we systematically evaluated the effects of three electrode surface activation techniques—alcohol activation, ECC, and ECR—on achieving the highest sensitivities toward H_2_O_2_, GABA, and GLU detection in an *in vitro* setting. We utilized a commercially available R1-Pt MEA (CenMET, Lexington, KY, USA) designed with four independent recording sites, each featuring an electrode area of 150 μm × 50 μm with a 50 μm spacing between microelectrodes, constructed from Pt deposited on a ceramic substrate (Figure 1a). This configuration features a MEA channel platform, ideal for localized NT measurements, making it well-suited for *in vivo* applications. We employed various techniques, including scanning electron microscopy (SEM), Raman spectroscopy, electrochemical impedance spectroscopy (EIS), cyclic voltammetry (CV), and amperometry, to investigate the impacts of square wave ECR pulses (+1.4 V, −0.25 V) at different frequencies (150–6,000 Hz) on the Pt microelectrode surface morphology, composition, chemistry, defects, and overall electrochemical characteristics. The findings of this study emphasize the crucial role of ECR parameters in significantly enhancing the sensitivity of Pt MEA-based GABA and GLU biosensors, providing a pathway toward ultrasensitive NT monitoring.

## 2 Experimental

### 2.1 Reagents and chemicals

All chemicals used in this study are reagent grade and were utilized as received. They were sourced from Sigma-Aldrich (St. Louis, MO, USA). The enzymes L-Glutamate oxidase (YMS-80049), a recombinant lyophilized powder (9.3 U/mg), and GABASE (G7509) were obtained from Cosmo Bio Co., Ltd. Deionized (DI) water was prepared using a three-stage filtration system (Modulab DI Recirculator, service deionization polisher) from Continental Water Systems.

### 2.2 Measurements and characterizations

Alcohol treatment was performed by soaking the R1-Pt MEA biosensor (CenMET, Lexington, KY, USA) in acetone and isopropyl alcohol for 5 min. This was followed by immersion in deionized (DI) water for 5 min, stirred at 200 rpm using a 1-inch magnetic stir bar. The R1-Pt MEA was then cleaned with a cotton swab. Also, ECC treatment was performed by soaking R1-Pt MEA in a 0.05 M H_2_SO_4_ electrolyte solution within a two-electrode system, using a saturated calomel electrode (SCE) (Gamry Instruments, Warminster, PA, USA) as the reference electrode and the R1-Pt MEA as the WE. The electrode was cycled between −0.3 V and +1.0 V for 15 cycles at 20 mV/s using cyclic voltammetry (CV) on a Gamry potentiostat (Gamry Instruments, Warminster, PA, USA) (Billa et al., 2022).

For ECR, the Owon AG4121 Single Channel Arbitrary Waveform Generator (Test Equipment Depot, Woburn, MA, USA) and oscilloscope (Tektronix Inc., Beaverton, OR, USA) were used. The ECR experiment was conducted in a 50 mL solution of 0.5 M perchloric acid, in a two-electrode setup with SCE as the reference electrode and R1-Pt MEA as the WE. The solution was stirred at 200 rpm with a 1″ magnetic stir bar, and the electrodes were exposed to +1.4 V/−0.25 V pulses at varying frequencies for 100 s. Afterward, a chronoamperometric cathodic bias of −0.2 V was applied for 180 s (Tan et al., 2022).

Electrochemical impedance spectroscopy (EIS) and cyclic voltammetry (CV) characterizations were performed using an AutoLab potentiostat (PGSTAT 302 N, Metrohm USA, Riverview, FL, USA). The measurements were conducted in a freshly prepared 50 mL solution of 5 mM [Fe(CN)₆]^3−^/^4−^ in 1 M KCl using a three-electrode system, with a SCE as the reference electrode, a 1 mm diameter platinum wire (Sigma-Aldrich, USA) as the counter electrode, and the R1-Pt MEA as the WE. CV was performed at a scan rate of 100 mV/s within a potential window of −0.3 V to +0.8 V, while EIS measurements were carried out with a 10 mV amplitude over a frequency range of 100 kHz to 0.1 Hz at open-circuit potential (OCP) (Tan et al., 2022).

Amperometric measurements were conducted using a four-channel FAST16mkII potentiostat system (Quanteon, LLC., Nicholasville, KY, USA) in a two-electrode configuration, with the R1-Pt MEA as the WE. An Ag/AgCl reference electrode is prepared by coating AgCl onto a 200-micron Ag wire (A-M Systems, Sequim, WA, USA) using a 5 M NaCl solution in 1 M HCl at 9 V for 1,200 s, employing the chronoamperometry technique.

Surface characterization was conducted using a Keyence VK-X200 Confocal Microscope, Raman spectroscopy, and a Hitachi S-4800 Scanning Electron Microscope (SEM). Raman spectroscopy (Control Development 2DMPP with *λ*: 514 nm). Sensitivity (SS, nAμM^−1^ cm^−2^) was determined from the slope of the current density (pA/μM) in the concentration plot within the linear range, normalized to the electrode area (7.5 × 10^−5^ cm^2^) (Tan et al., 2022). Data analysis and plotting were performed using OriginPro (Version 2025, OriginLab Corporation, USA) and Microsoft Excel.

### 2.3 Enzyme coating procedure

The enzyme matrix solution was freshly prepared by mixing 1% BSA and 0.125% glutaraldehyde in DI water. This matrix solution was then combined with GOx (0.1 U/μL) and GABASE (0.1 U/μL) in an 8:1:1 ratio, following the detailed procedure described in (Hossain et al., 2018; Moldovan et al., 2021). A 0.02 μL drop of the freshly prepared, respective enzyme solution was precisely drop-cast onto each Pt microelectrode surface using a 0.05 μL Hamilton syringe (Hamilton Company, Reno, NV, USA) under a Keyence confocal microscope (Keyence Corporation, Osaka, Japan). The coated biosensor was wrapped in aluminum foil and stored in a refrigerator for 48 h to ensure complete crosslinking. The biosensor performance was evaluated in a 50 mL beaker containing 1X PBS and a freshly prepared 100 μM *α*-ketoglutarate solution, stirred at 200 rpm in a water bath chamber at room temperature using the FAST16mkII system (Hossain et al., 2018).

### 2.4 Amperometry calibration and detection of NTs

Amperometry measurements were conducted in a freshly prepared 50 mL 1X PBS solution (pH 7.4) that was continuously stirred at 200 rpm with a 1-inch magnetic stir bar at room temperature inside a water bath chamber. A 2-electrode setup was used, with the Pt MEA as the WE and a Teflon-coated Ag/AgCl wire as the reference electrode that was connected to the FAST16mkI system (Moldovan et al., 2021). For H₂O_2_ calibration studies, varying concentrations of H_2_O_2_ (2–10 μM) were injected at 1-min intervals using the chronoamperometric technique at a fixed potential of +0.7 V (Hossain et al., 2018). GABA calibration studies were conducted by injecting varying concentrations of GABA (1–40 μM) into the freshly prepared 1X PBS solution containing 100 μM α-ketoglutarate. Similarly, GLU calibration was performed by injecting varying concentrations of GLU (5–40 μM). In both cases, injections were made at 1-min intervals using a chronoamperometric technique at a fixed potential of +0.7 V, representing the expected GABA and GLU concentrations in the animal models (Hossain et al., 2018). All measurements were repeated (*n* = 3), and the data were analyzed using FAST Analysis® software.

## 3 Results and discussion

### 3.1 Effect of surface activation on Pt microelectrode surface properties

Scanning electron microscopy (SEM) images (Figures 1b–k) reveal significant changes in the electrode surface morphology “texture,” with a noticeable increase in surface roughness and porosity, particularly heightened during the ECR treatments performed at moderate frequencies (2,500 Hz). After alcohol and ECC treatments, the surface was relatively smooth and clean. However, the ECR treatments induced significant recrystallization, grain growth, and particle agglomeration, resulting in a highly heterogeneous surface with varying levels of porosity and enhanced surface roughness. No visible signs of electrode delamination, structural damage, cracking, or fouling were detected, confirming the utility and safety of the ECR treatment in roughening Pt thin film microelectrodes while preserving the film integrity. This preservation of structural integrity is critical for maintaining sensor durability and performance during electrochemical measurements. To quantitatively assess the changes in surface roughness, a Keyence Laser Confocal Microscope was employed. The average surface roughness (*R_a_*) for the alcohol-treated surface is 0.001 ± 0.0001 μm, indicating a very smooth surface, whereas it is 0.20 ± 0.01 μm for the ECR-treated surface. This enhancement is expected to increase the electroactive surface area and improve sensor sensitivity, as confirmed by CV and EIS measurements (discussed in the following two sections). We employed Raman spectroscopy to evaluate the role of carbon (graphene) film deposition, its presence, and interference on the electrochemical behavior after ECC and ECR surface activation treatments (refer to Supplementary Figure S1; Supplementary Table S1). The G band is the primary mode in the Raman spectrum of graphene, which is used to evaluate the thickness and quality of graphene layers. It is a sharp band at approximately 1,585 cm^−1^, representing in-plane stretching vibrations within the *sp^2^*-bonded hexagonal lattice. The position and intensity of this band are indicative of the number of layers present. As the number of layers in a carbon film increases, the Raman shift of the G band decreases, and the intensity increases. The D band, at approximately 1,350 cm^−1^, is only observed when defects exist in the layers and serves as a key indicator of graphene quality. The intensity of the D band in a spectrum is directly correlated to the amount of disorder in the region probed. Post-alcohol and ECC-treated surfaces exhibited a relatively small presence of carbon film. In contrast, the ECR treatment resulted in observable G and D bands. The 250 Hz ECR treatment yielded the lowest I_D_/I_G_ ratio (0.503), indicating the formation of a highly ordered graphitic carbon film. The 2,500 Hz ECR sample exhibited the narrowest D-band (122.44 cm^−1^) with a slightly increased I_D_/I_G_ ratio (0.532), indicating well-defined defect domains within the carbon layer and, importantly, showed the lowest absolute Raman intensities, implying the carbon film is comprised of the least number of graphene layers. The 4,000 Hz ECR treatment displayed the highest I_D_/I_G_ ratio (0.803), suggesting the deposition of significantly disordered carbon. The 6,000 Hz ECR treatment exhibited the highest absolute Raman intensities, corresponding to the most significant amount of carbon deposition and an I_D_/I_G_ ratio of 0.685. As discussed in the following sections, the H_2_O_2_ sensitivity peaked at 2500 Hz, indicating that the ideal ECR-treated Pt surface should have well-defined defect domains with the least number of graphene layers. Thus, the carbon appears to act primarily as an impurity in this work.

### 3.2 Effect of surface activation techniques on H_2_O_2_ sensitivity

#### 3.2.1 Alcohol treatment

**Figure 2:**
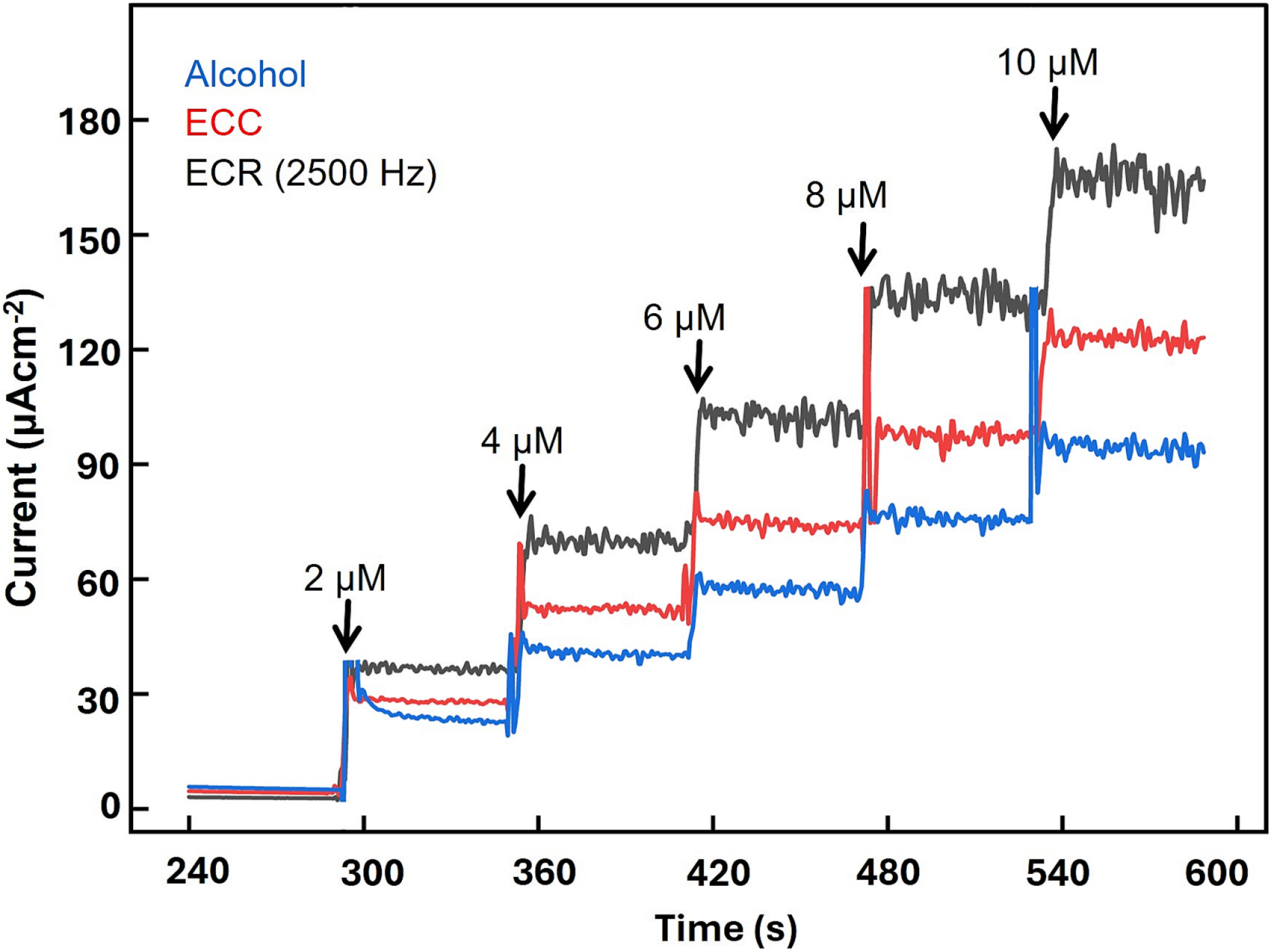
Effect of the surface activation techniques on H₂O_2_ oxidation currents. The H_2_O_2_ concentration was varied between 2.0 μM and 10 μM. Amperometry measurements were conducted at +0.7 V vs. Ag/AgCl in a 50 mL beaker containing 1X PBS, stirred at 200 rpm, and maintained at 37 °C.

For alcohol treatment, the Pt microelectrode was sequentially soaked in acetone and isopropyl alcohol for 5 min each, followed by a thorough rinse with DI water. Subsequently, the microelectrode surface was gently cleaned using a cotton swab to remove any organic residues and contaminants from the MEA fabrication process and any adventitious carbonaceous material that may interfere with the microelectrode’s electrochemical response. The effectiveness of the treatment was assessed by evaluating the electrode’s sensitivity to H₂O_2_, the electroactive by-product of GABA and GLU biosensors. The alcohol-treated microelectrodes demonstrated an enhanced sensitivity of 2,502 ± 144 nA μM^−1^ cm^−2^ (*n* = 3) (Figure 2, blue curve), representing a 39% increase compared to the no-cleaning control of 1,800 ± 120 nA μM^−1^ cm^−2^ (*n* = 3). This improvement can be attributed to the removal of surface impurities and the exposure of active platinum sites, which promote better electron transfer kinetics and amplify the detection signal, as confirmed by CV and EIS measurements (details in the following sections).

#### 3.2.2 ECC treatment

Following the initial alcohol treatment, the Pt MEAs underwent an additional ECC procedure to enhance their performance further. The ECC was conducted by applying a cyclic voltage sweep within a potential window of −0.3 V and +1.0 V for 15 cycles at a scan rate of 20 mV/s using a 0.05 M H₂SO_4_ electrolyte solution, as described in (Doughty et al., 2020). This resulted in the effective removal of strongly adherent residual contaminants, facilitating their desorption and regenerating the Pt surface, thereby restoring its electrocatalytic activity. ECC also promoted the formation of an active platinum oxide layer, which enhanced electron transfer kinetics and improved the electrode’s responsiveness to target analytes. As demonstrated in Figure 2, the red curve shows that ECC treatment significantly enhances the electrode’s sensitivity to H_2_O_2_, a key indicator of enzymatic activity in biosensing applications. The sensitivity increased to 4,046 ± 151 nA μM^−1^ cm^−2^, a 61.7% increase compared to the alcohol treatment, which is again due to a marked improvement in the electroactive surface area, the removal of surface impurities, and/or changes to the surface chemistry and functional groups.

#### 3.2.3 ECR treatment

**Figure 3:**
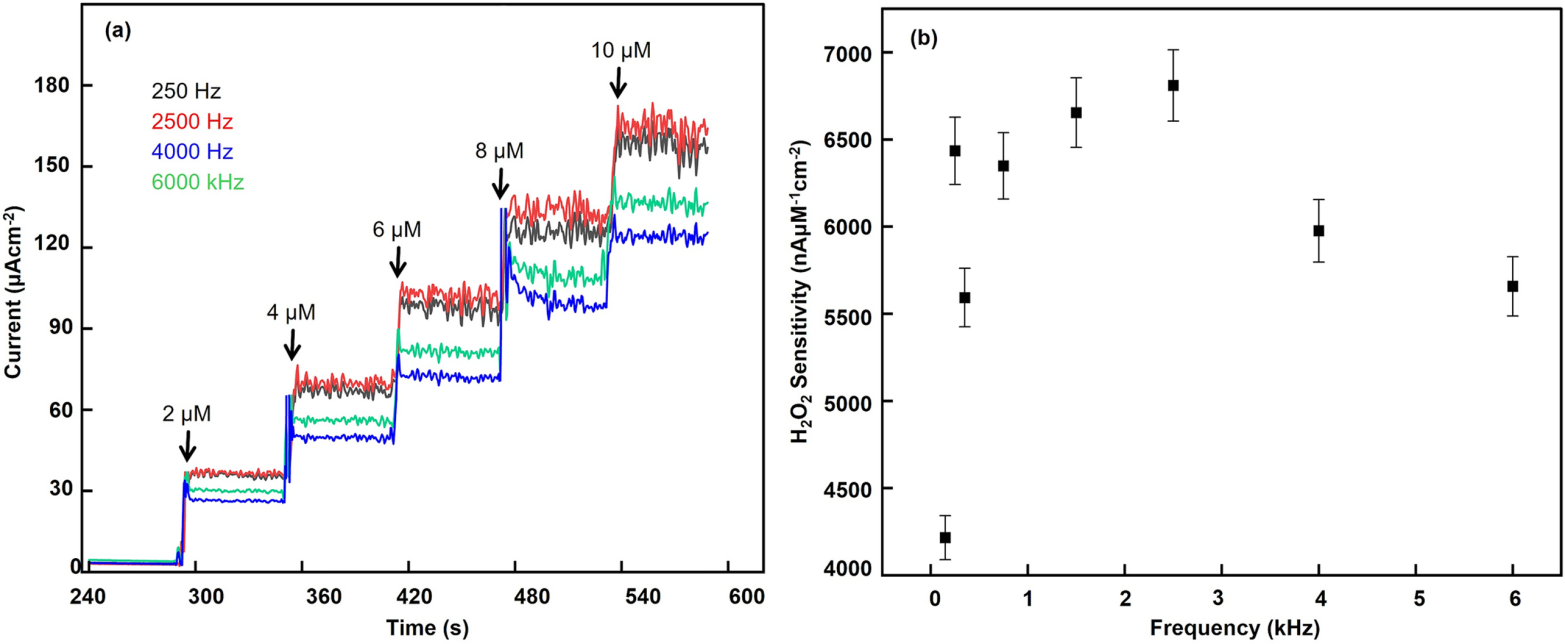
Effect of ECR pulse frequency on H_2_O_2_ oxidation currents and sensitivity **(a)** Calibration curves at 250, 2,500, 4,000, and 6,000 Hz at a +1.4 V/−0.25 V ECR pulse parameters. **(b)** Sensitivity plots at 150, 250, 350, 750, 1,500, 2,500, 4,000, and 6,000 kHz, respectively. The H_2_O_2_ concentration was varied between 2 μM and 10 μM (*n* = 3). Amperometry measurements were conducted at +0.7 V vs. Ag/AgCl in a 50 mL beaker containing 1X PBS, stirred at 200 rpm, and maintained at 37 °C.

To further enhance the H_2_O_2_ sensitivity values, the microelectrodes were treated with the ECR process, as outlined in (Tan et al., 2022; Figure 2, black curve). The effect of ECR on the microelectrode’s sensitivity to H₂O_2_ was evaluated by applying a square wave potential at +1.4 V/−0.25 V across a range of frequencies (150, 250, 350, 750, 1,500, 2,500, 4,000, 6,000 Hz). The objective here is to identify the optimal frequency that maximizes surface roughening, enhances the electroactive area, and improves electrocatalytic activity without causing any surface damage or delamination (Figures 1d–k). The ECR treatment significantly roughened the Pt surface by creating a highly porous surface. The most notable enhancement in sensitivity was observed at a frequency of 2,500 Hz, yielding a considerably improved H_2_O_2_ sensitivity value of 6,810 ± 103 nA μM^−1^ cm^−2^ (Figures 3a,b), a 68.3% increase compared to ECC treatment. This is the highest H_2_O_2_ sensitivity reported in the literature for a porous Pt microelectrode as compared to 5,873 ± 283 nA μM^−1^ cm^−2^ reported by (Tan et al., 2022). This improvement is attributed to achieving a suitable mixture of flat and porous regions with optimal geometry, size, and conductivity, resulting in a maximum electroactive area for H_2_O_2_ adsorption and improved electrocatalytic properties, as supported by CV and EIS studies (details in the following section).

### 3.3 Effect of surface activation techniques on electrochemical characteristics

#### 3.3.1 Cyclic voltammetry (CV) studies

**Figure 4:**
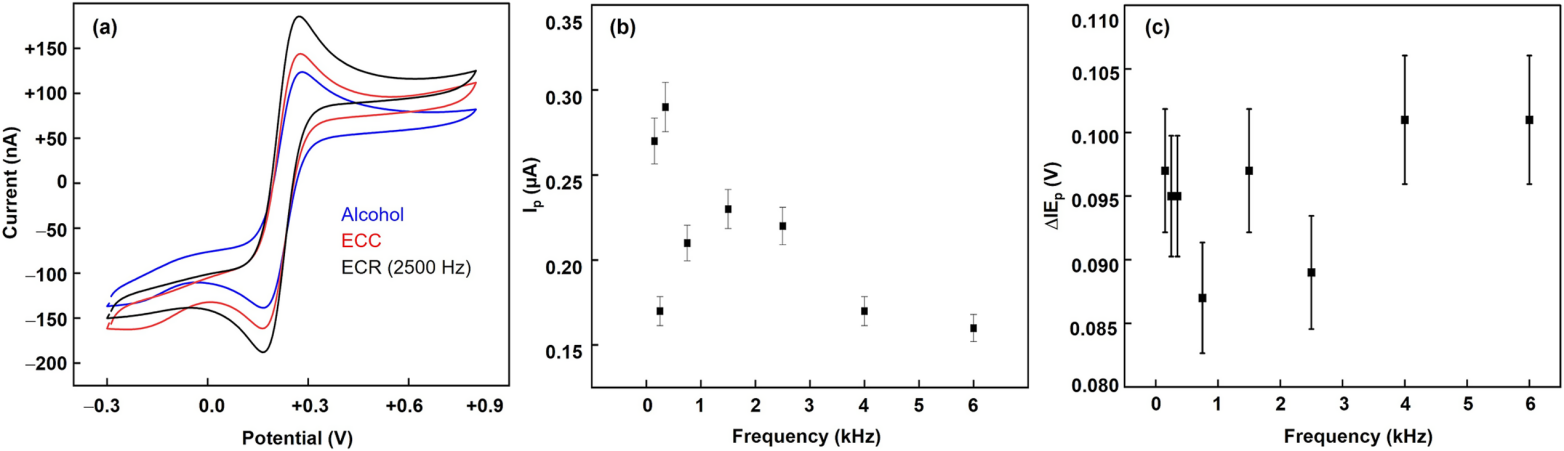
**(a)** Effect of surface activation techniques on the cyclic voltammogram behavior of treated Pt microelectrodes. **(b,c)** The effect of ECR pulse frequency (150, 250, 350, 750, 1,500, 2,500, 4,000, and 6,000 Hz) on the forward peak currents and the ΔE peak separations of treated Pt microelectrodes at a +1.4 V/−0.25 V ECR pulse parameters. A 5 mM [Fe(CN)_6_]^3−/4−^ solution in 1 M KCl was used in a 3-electrode setup. The reference is a saturated calomel electrode, and the counter electrode is a 1 mm diameter Pt wire. The potential is cycled between −0.3 V and +0.8 V at a scan rate of 100 mV/s.

We utilized the CV technique to investigate the effect of the three treatments on the electroactive area and electrode kinetics. Figure 4 illustrates a significant increase in the electroactive surface area following ECR treatment. The peak current (Iₚ) was used as a direct indicator of the electroactive area, with a significant increase observed for the ECR-treated electrodes compared to the alcohol and ECC-treated surfaces. Specifically, the ECR-treated electrode exhibited a peak current of 0.22 ± 0.01 μA, representing a 38% increase compared to the alcohol-treated surface. The ECC-treated surface reached a peak current of 0.18 ± 0.02 μA, demonstrating a moderate enhancement compared to the alcohol treatment. The alcohol-treated surface displayed a peak current of 0.16 ± 0.02 μA, representing the baseline with minimal surface activation (Figure 4a). Among the ECR frequencies, 250 Hz yielded the highest oxidation peak currents and electroactive area, but the E_p_ value is also higher (~95 mV), indicating slower kinetics and electrocatalytic activity (Figure 4b). At 2500 Hz, though the peak oxidation current is lower than that of a 250 Hz-treated microelectrode, the ΔE_p_ value is lower (~88 mV), suggesting facile kinetics and higher electrocatalytic activity. These findings highlight the effectiveness of the ECR technique and, importantly, the application of optimal pulse frequency in enhancing the electrode’s surface and electrochemical properties, leading to enhanced H_2_O_2_ sensitivity.

#### 3.3.2 Frequency-dependent electrochemical interfacial properties

We performed a detailed EIS analysis to understand the effect of ECR treatment on the sensitivity to H_2_O_2_, GABA, and GLU. The EIS circuit fitting of alcohol-treated microelectrodes reveals two distinct regions on the surface: Region 1, comprising nano-sized Pt grains that form a smooth surface, and Region 2, characterized by grain boundaries with an irregular, pore-like structure. This porous region is the space between the Pt nanocrystals, filled with electrolyte, as shown in the SEM images. The ECC treatment removed the adsorbed layer, as indicated by the EIS data fitting. Although regions 1 and 2 remain on the electrode surface, region 1 has flattened, reducing the charge transfer resistance (*R_ct_*) by about threefold and decreasing the capacitive value by fourfold due to less grain charging and changes in surface functional groups. This suggests a slight decrease in the conductivity of region 1. In contrast, the conductivity of region 2 (the porous region) has improved by approximately 20%. Overall, ECC treatment leads to the removal of the adsorbed layer, a slight decrease in conductivity in region 1, and a marginal increase in region 2. In addition, comparing the EIS data at a frequency of 1,098 Hz between the two treatments (alcohol and ECC) suggests that the overall impedance of the electrodes has been reduced approximately by half after ECC treatment. A shift in the data for ECC-treated electrodes is observed at approximately 1 kHz (1,098 Hz) (refer to Supplementary Figure S2). At this frequency, the impedance of ECC-treated electrodes starts to decrease. Thus, this frequency is considered for comparison purposes.

**Figure 5:**
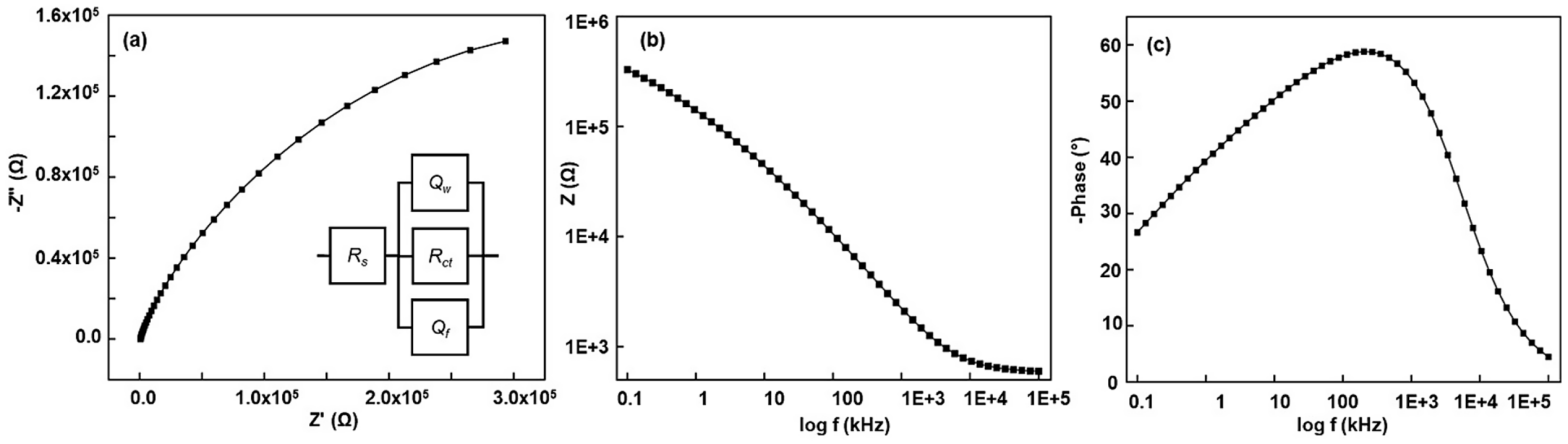
**(a–c)** Representative Nyquist, Bode modulus, and Bode phase plots of ECR-treated Pt microelectrode at 2500 Hz pulse frequency. Inset in **(a)** corresponds to the equivalent circuit model. EIS was carried out at a 10-mV amplitude and frequencies ranging from 0.1 Hz to 100 kHz. A 5 mM [Fe(CN)_6_]^3−/4−^ solution in 1 M KCl was used in a 3-electrode setup. The reference is a saturated calomel electrode, and the counter electrode is a 1 mm diameter Pt wire.

Next, for ECR-treated microelectrodes, the Nyquist plot showed a significant change in the impedance spectra, specifically a transition from a hump at higher frequencies, followed by an arc to no hump and a symmetrical arc (Figure 5a), and the Bode phase plot now comprises a single broad peak (Figure 5c). The surface is now microstructurally uniform, without distinct regions. It features heterogeneous pores that operate as a single system, each with unique geometry and size. The fitted circuit represents an irregular pore; the first circuit element is *Q_W_*, a constant phase element of the walls of the pores whose N_w_ value varies between 0.5 or lower; the second element is *Q_f_*, a constant phase element representing the heterogenized flat surface between the pores whose N_f_ value varies from 1 to 0.8, the third circuit element is *R_ctp_*, the charge transfer resistance inside the pore, between walls and the electroactive species, and the fourth element is *R_s_*, the solution resistance outside of the pore. The first three circuit elements form a parallel circuit. This is mainly because each element acts as an independent unit yet is connected.

As observed in SEM images (Figures 1d–k), the heterogeneity in region 1 arises due to the recrystallization (fractalization) of the Pt grains. Table 1 indicates that grain fractalization (heterogeneity) occurs most frequently at lower frequencies (150–350 Hz), resulting in increased pore conductivity due to the more heterogeneous, flat surface and pore walls. Notably, electrodes treated with a 250 Hz waveform exhibit more conductive pore walls than those treated with 150 Hz and 350 Hz, while their flat surfaces show similar conductivity. In contrast, higher frequencies (750–2,500 Hz) reduce fractalization, as they lack the impulse (force times the period of the signal) required for significant fractalization. Consequently, at 2500 Hz, the flat surface remains smooth with higher impedance compared to heterogeneous grains. Overall, lower-frequency treatments enhance the electrode’s conductivity more than higher-frequency treatments, as shown in Table 1, generating more catalytically active sites. This new understanding is further validated by the impedance values of the Bode modulus obtained at a frequency of 1,098 Hz (Figure 5b).

**Table 1:** The effect of frequencies on the typical EIS circuit values of the elements of the ECR-treated Pt microelectrodes and the H_2_O_2_ sensitivity.

Frequency (Hz)	R_s_ (*Ω*)	Q_f_ (nMho), N_f_	Q_W_ (μMho), Nw	R_ctp_ (kΩ)	Bode modulus (Ω) measured at 1 kHz	Y_f_ (F. f^1-N^/cm^2^)	Y_W_ (F. f^1-N^/cm^2^)	H_2_O_2_ sensitivity (nAμM^−1^cm^−2^)
150	575	380 *N* = 0.846	2.53 *N* = 0.394	494	1,597–2,632	1.92	5.32 × 10^−1^	4,216 ± 88
250	621	500 *N* = 0.818	2.44 *N* = 0.41	769	1,603–1,535	2.04	5.74 × 10^−1^	6,435 ± 510
350	571	583 *N* = 0.801	2.67 *N* = 0.33	543	1,622–1,700	2.12	3.27 × 10^−1^	5,593 ± 89
750	560	146 *N* = 0.819	1.87 *N* = 0.492	707	3,256–3,505	6.01 × 10^−1^	7.8 × 10^−1^	6,349 ± 32
1,500	625	40.1 *N* = 0.928	1.76 *N* = 0.511	702	3,738–3,840	3.5 × 10^−1^	8.39 × 10^−1^	6,654 ± 68
2,500	543	5.12 *N* = 1	2.45 0.484	910	5,350–5,394	7.5 × 10^−2^	9.68 × 10^−1^	6,810 ± 124

For EIS, the percentage errors for the circuit elements are below 14% (*n* = 3).

The H_2_O_2_ calibration data suggest that 2,500 Hz ECR treatment leads to the highest sensitivity. This could be because the unique geometry, size, and conductivity of the pore walls, as well as the absence of carbon (refer to Supplementary Figure S1; Supplementary Table S1), played a more significant role in H_2_O_2_ adsorption than the flat surface region of the microelectrode. It is observed that the treatments of 2,500 Hz, 1,500 Hz, 750 Hz, and 250 Hz similarly carve or fractalize the pore walls (in terms of geometry and size), as suggested by their similar N_w_ values, between 0.41 and higher, so that the walls have similar geometries, thus resulting in similar H_2_O_2_ sensitivities. Furthermore, we calculated the admittance of the flat surface (Y_f_) and pore walls (Y_w_) using the circuit values of their respective constant phase elements and compared it with H_2_O_2_ sensitivity (Table 1). The data indicate that roughening the microelectrode at lower frequencies enhances conductivity, with 250 Hz showing the highest sensitivity due to its combination of a heterogeneous flat surface and highly conductive pore walls. Each pulse uniquely modifies the Pt surface, and the conductivity of the pore walls increases with frequency. Among treatments from 750 Hz to 2,500 Hz, 2,500 Hz has the highest sensitivity but lacks carbon in the pores, as indicated by Raman data. The flat surface of the 250 Hz-treated electrode is 27 times more conductive than that of the 2,500 Hz-treated electrode. While the pore walls of the 2,500 Hz-treated electrodes are twice as conductive as those at 250 Hz, this does not translate to a proportional increase in H_2_O_2_ sensitivity, possibly due to stagnant solution effects limiting the diffusion of fresh electroactive species. The flat surface shows the lowest conductivity due to oxidized Pt. The N values of the electrodes reveal a similar trend, indicating that high-frequency treatment does not create distinct pores. Although the 2,500 Hz electrode has a smaller electroactive area than the 250 Hz electrode, its pore geometry, conductive walls, and lower carbon content enhance Pt-induced catalytic activity of H_2_O_2_. The conductivity, geometry, and size of pore walls are crucial for H_2_O_2_ adsorption and sensitivity. Mild oxidation from ECR is not always reversible during normal cycling and may result in permanent deactivation of the electrode surface. ECC can partially recover Pt’s electrocatalytic activity, but prolonged oxidation degrades performance. This analysis suggests that the highest sensitivity is achieved through the absence of carbon, the presence of smooth, pristine Pt grains, and the highly conductive nature of the heterogeneous pore walls, rather than solely from a larger electroactive electrode area. Additionally, it is possible that electrodes featuring a larger electroactive area, a specific pore geometry that enhances diffusion within the pores, highly conductive pore walls, and a low carbon impurity content could ultimately lead to even greater H_2_O_2_ sensitivity.

### 3.4 Effect of surface activation techniques on GABA and GLU sensitivity and LODs

**Figure 6:**
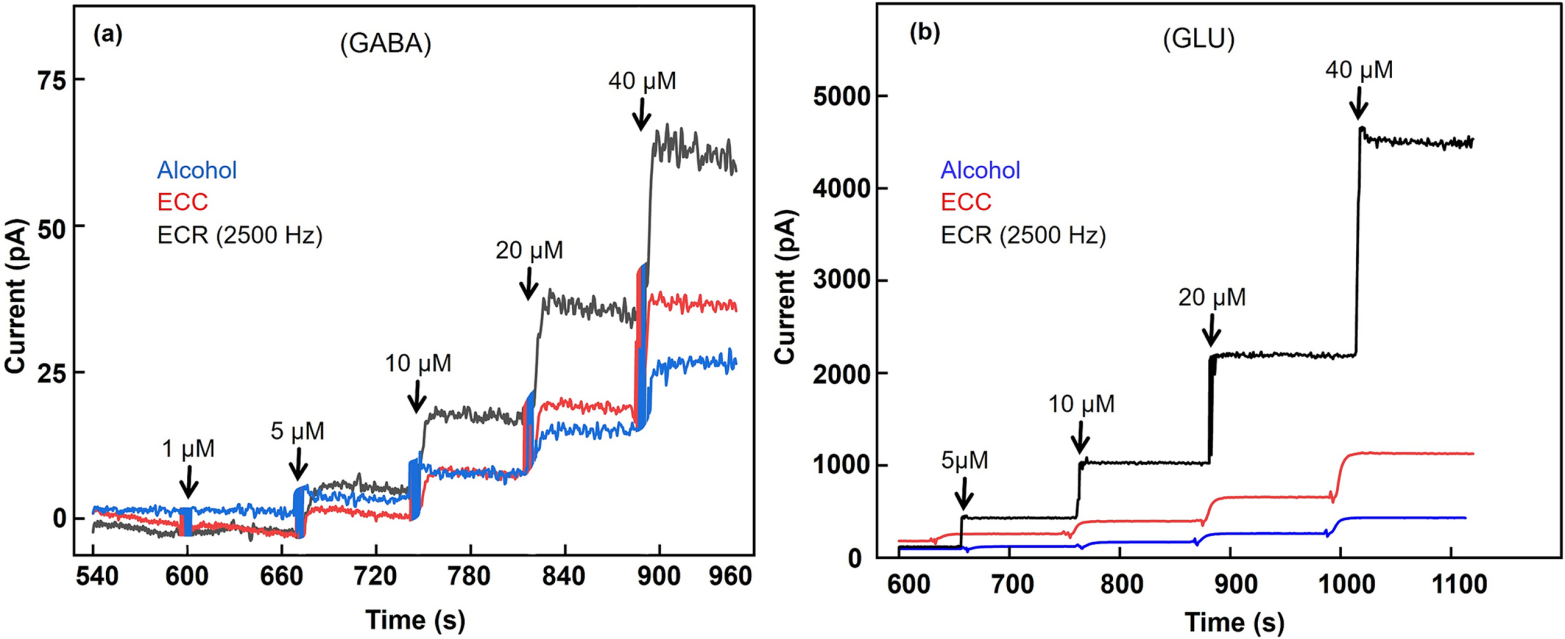
Effect of surface activation techniques on GABA and GLU responses. **(a)** *In vitro* calibration of GABA (1, 5, 10, 20, 40 μM) **(b)** *In vitro* calibration of GLU (5, 10, 20, 40 μM). The experiments were conducted in 100 μM *α*-ketoglutarate prepared in 1X PBS buffer. The Pt microelectrodes were biased at +0.7 V vs. Ag/AgCl reference while stirring the solution at 200 rpm.

Following alcohol treatment, the GABA and GLU sensitivities were 7.00 ± 0.6 nA μM^−1^ cm^−2^ and 182.20 ± 4.9 nA μM^−1^ cm^−2^ (*n* = 3), respectively. Post-ECC treatment increased these values to 12 ± 0.3 nA μM^−1^ cm^−2^ and 520 ± 12.3 nA μM^−1^ cm^−2^ for GABA and GLU (*n* = 3). Following the ECR treatment at the optimal applied frequency of 2,500 Hz, the biosensors exhibited a remarkable increase in the sensitivity values, achieving 45 ± 4.4 nA μM^−1^ cm^−2^ and 1,510 ± 47.0 nA μM^−1^ cm^−2^ for GABA and GLU, respectively (Figures 6a,b; Table 2). This represents a ~ 3-fold increase in both GABA and GLU sensitivity as compared to ECC treatment. The corresponding limits of detection are 1.60 ± 0.13 nM and 12.70 ± 1.73 nM (*n* = 3). These enhanced sensitivity values notably exceed those reported in (Tan et al., 2022; Ivanovskaya et al., 2018; Moldovan et al., 2021), highlighting the effectiveness of the ECR treatment in enhancing NT detection. The substantial gains in GABA and GLU sensitivity suggest improved surface reactivity and electron transfer efficiency, positioning this electrode surface activation approach as a promising advancement for ultrasensitive GABA and GLU sensing.

**Table 2:** Effect of surface activation techniques on the sensitivities of H_2_O_2_, GABA, and GLU (*n* = 3).

Surface activation technique	H_2_O_2_ sensitivity (nAμM^−1^ cm^−2^)	GABA (nAμM^−1^ cm^−2^)	GLU (nAμM^−1^ cm^−2^)
Alcohol-treated	2,502 ± 144	7 ± 0.6	182 ± 4.9
ECC-treated	4,046 ± 151	12 ± 0.3	520 ± 12.3
ECR (2,500 Hz)-treated	6,810 ± 124	45 ± 4.4	1,510 ± 47.0

To evaluate the reproducibility of our findings, we fabricated three independent batches of ECR-treated R1-Pt MEAs and measured the percentage Relative Standard Deviation (%RSD) for sensitivities to H_2_O_2_, GABA, and GLU. The %RSD values obtained were 1.24, 4.73, and 3.50%, respectively, indicating high reproducibility across the electrode batches. To achieve selectivity, long-term stability, and fouling resistance, studies essential for *in vivo* applications, the GABA and GLU biosensors will be coated with 1,3-phenylenediamine (mPD), a size-exclusion coating, widely used in this field (Billa et al., 2022; Doughty et al., 2020; Tan et al., 2022). Previous findings from our group and others indicate that mPD coatings exhibit sufficient selectivity *in vitro* during 2-month stability studies and *in vivo* for up to 11 days against common interferents, including ascorbic acid, uric acid, dopamine, and serotonin, at concentrations of up to 200 μM. Thus, we expect an 80 μm thick mPD to be adequate for short-term *in vivo* applications, up to 2 weeks.

## 4 Conclusion

ECR-treated Pt microelectrodes significantly enhance the sensitivity of GABA and GLU biosensors. SEM, EDS, Raman, CV, and EIS analyses reveal a microstructure with heterogeneous pores that work together, each with unique geometry and size. At lower ECR frequencies (150–350 Hz), fractalization of Pt nanocrystals increases heterogeneity and pore conductivity, leading to more carbon impurity deposition. At higher frequencies (750–2,500 Hz), smooth grains in flat electrode regions exhibit increased impedance and reduced conductivity due to oxidized platinum, whereas pore walls maintain high conductivity with minimal carbon impurities. Achieving an optimal balance of flat and porous regions is essential for maximizing the electroactive area for H_2_O_2_ adsorption, enhancing electrocatalytic properties, and achieving high detection sensitivities. This work highlights that ECR parameters, pore structure, surface functionalities, and impurities influence the properties of a new class of Pt microelectrodes. The ECR approach is adaptable for other enzymatic (oxidase) biosensors, particularly those utilizing metallic surfaces, such as platinum, with a focus on H_2_O_2_ detection (e.g., biosensors for choline, acetylcholine, lactate, glucose, and cholesterol). Key advantages of ECR-treated Pt MEA biosensors include significant sensitivity improvements for H_2_O_2_ (6,810 ± 124.0 nA μM^−1^ cm^−2^), GABA (45 ± 4.4 nA μM^−1^ cm^−2^), and GLU (1,510 ± 47.0 nA μM^−1^ cm^−2^) at 2,500 Hz pulse frequency, promising advancements in NT biosensors for chemical neuroscience. The straightforward, scalable ECR process enables selective activation of Pt microelectrodes for developing ultrasensitive biosensors. Additionally, the established EIS model supports the design of compact enzymatic biosensors for enhanced NT detection, which is crucial for *in vivo* behavioral studies, thereby paving the way for ultra-miniaturized MEAs suitable for neurological disorder research and clinical diagnostics.

## Data Availability

The original contributions presented in the study are included in the article/Supplementary material, further inquiries can be directed to the corresponding author.
