# Supplementary material for: Enhancing the sensitivity of gamma-aminobutyric acid and glutamate biosensors by electrochemically roughening platinum microelectrodes
Source: Front Neurosci. 2025 Sep 19;19:1679591. doi: 10.3389/fnins.2025.1679591 (PMC12491176; doi:10.3389/fnins.2025.1679591)
Supplement: Supplementary file 1 [file Data_Sheet_1.DOCX]

Supporting Information for

**Enhancing the Sensitivity of Gamma-Aminobutyric Acid and Glutamate Biosensors by Electrochemically Roughening Platinum Microelectrodes**

*Musefiu Yemi Adediji ^1,^ Sanjeev Billa ^1^, Shabnam Siddiqui^2^, Prabhu U. Arumugam^1,2,*^*

^1^Institute for Micromanufacturing (IfM), Louisiana Tech University, Ruston, LA 71272

^2^Center for Biomedical Engineering and Rehabilitation Science (CBERS), Louisiana Tech University, Ruston, LA 71272

*** Correspondence:**Prabhu U. Arumugam
[parumug@latech.edu](mailto:parumug@latech.edu)

**
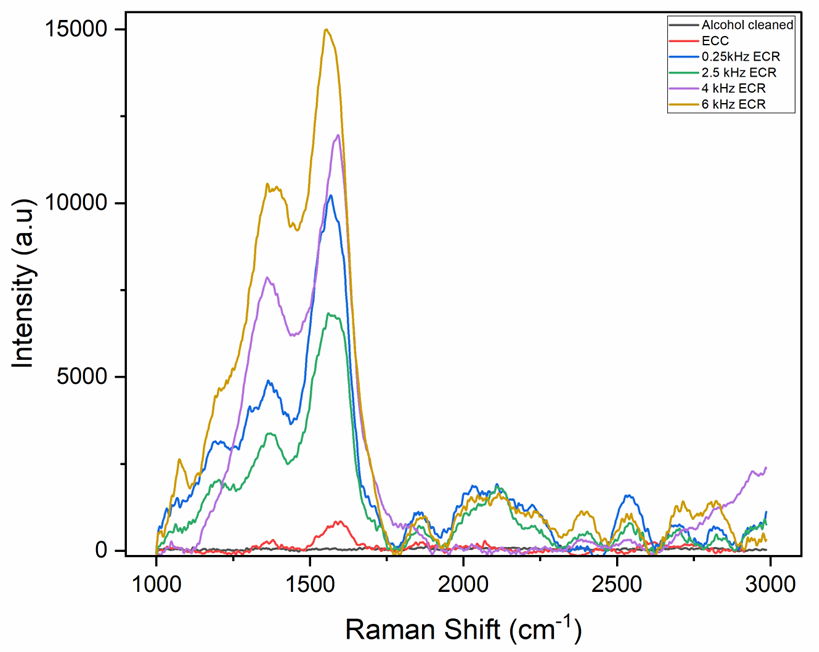
**

**Figure S1** shows Raman spectra comparing carbon deposits formed on platinum electrodes following different electrochemical treatments. The spectra display characteristic D-band (~1350 cm⁻¹) and G-band (~1580 cm⁻¹) peaks associated with carbon materials, with intensity varying by treatment method. Alcohol treated platinum (black line) shows minimal signal, while ECC and ECR at different frequencies (0.25-6 kHz) produce significant carbon deposits.

**Supplementary Figure 1**


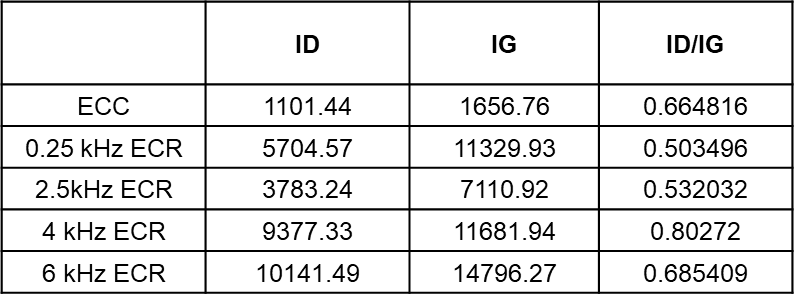


**Table S1:** This table quantifies peak intensities (I_D_, I_G_) and their ratios (I_D_/I_G_), revealing the effect of surface activation techniques on the deposited carbon structure and its disorder on the Pt electrode surface.


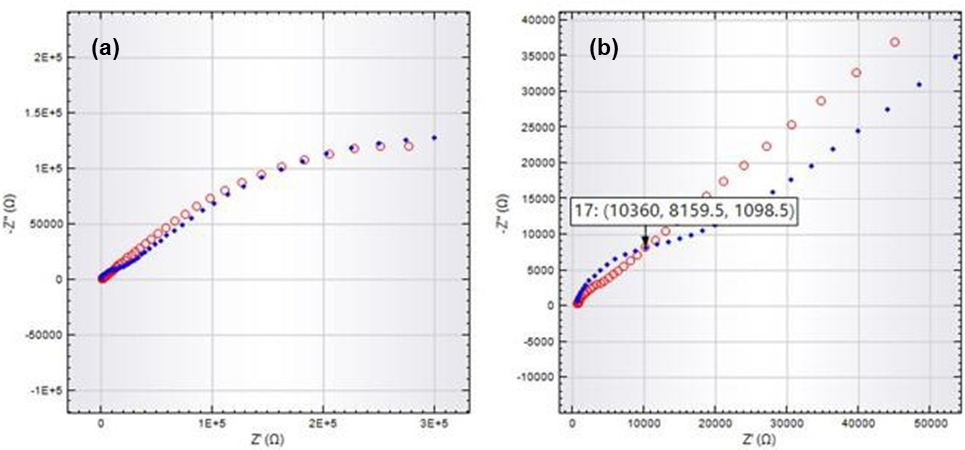

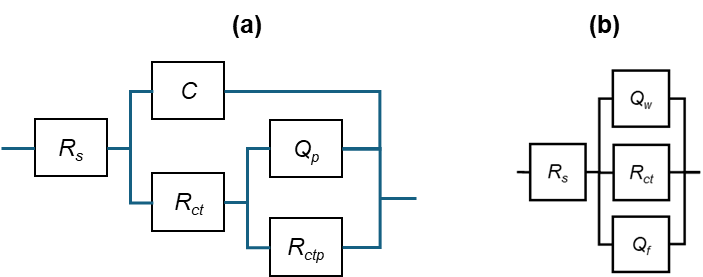


**Figure S2. (a)** A comparison of EIS data for the alcohol-treated (red curve) electrode with that of the ECC-treated (blue curve) electrode. (b) Expanded plot of (a), showing a reduction in impedance is observed in the data for ECC-treated electrodes at a frequency of approximately 1 kHz (1098 Hz).

**Supplementary Figure 2**

**Figure S3.** A comparison of the equivalent circuit modes before (**a**) and after (**b**) the ECR treatment of the Pt microelectrodes. For (**a**), the circuits consist of the following circuit elements: solution resistance (*R_S_*), capacitance (*C*), charge transfer resistance (*R_ct_*), constant phase element, and inhomogeneity factor of the porous region (*Q_p_*, *N_p_*), charge transfer resistance in the porous region (*R_ctp_*). For (**b**), the circuit represents an irregular pore; the first circuit element is *Q_W_*, a constant phase element of the walls of the pores whose Nw value varies between 0.5 or lower; the second element is *Q_f_*, a constant phase element representing the heterogenized flat surface between the pores whose N_f_ value varies from 1 to 0.8, the third circuit element is *R_ctp_*, the charge transfer resistance inside the pore, between walls and the electroactive species, and the fourth element is *R_s_*, the solution resistance outside of the pore.

**Supplementary Figure 3**
